# Recent Advances in Glutathione Depletion-Enhanced Porphyrin-Based nMOFs for Photodynamic Therapy

**DOI:** 10.3390/pharmaceutics17020244

**Published:** 2025-02-12

**Authors:** Bin Gong, Qiuyun Zhang, Jiayi Chen, Yijie Qu, Xuanxuan Luo, Weiqi Wang, Xiaohua Zheng

**Affiliations:** 1 The People’s Hospital of Danyang, Affiliated Danyang Hospital of Nantong University, Danyang 212300, China; 2 School of Pharmacy, Nantong University, Nantong 226001, China

**Keywords:** porphyrin, metal–organic frameworks, photodynamic therapy, singlet oxygen, glutathione depletion

## Abstract

Photodynamic therapy has established itself as a clinical treatment for certain superficial cancers by converting oxygen into cytotoxic singlet oxygen to eradicate cancer cells. Porphyrin-based nanoscale metal–organic frameworks have emerged as promising photosensitive platforms due to their ability to prevent the hydrophobic aggregation quenching of porphyrin molecules and enhance accumulation at the tumor site, thereby becoming a focal point in photodynamic materials research. However, the elevated levels of glutathione and other reductive substances within cancer cells can alleviate the oxidative stress induced by singlet oxygen from the photodynamic therapy process, thus protecting intracellular biomolecular structures from damage. Consequently, it is crucial to design functionalized nanoplatforms that integrate glutathione depletion with porphyrin-based metal–organic frameworks to significantly boost photodynamic therapy efficacy. Moreover, the excess glutathione within cells can disrupt the structure of porphyrin-based metal–organic frameworks, which not only increases the capacity of porphyrin molecules to generate singlet oxygen upon light exposure but also aids in the recovery of their fluorescence imaging capabilities. Additionally, this specificity minimizes the photosensitizing harm of porphyrin-based metal–organic frameworks to other normal tissues. This review compiles recent advancements in developing porphyrin-based metal–organic frameworks for enhanced phototherapy through glutathione depletion. It aims to promote the further application of porphyrin-based metal–organic frameworks in phototherapy and provide valuable insights for preclinical applications. By highlighting strategies that improve therapeutic outcomes while maintaining safety profiles, this summary seeks to advance the development of more effective and targeted cancer treatments.

## 1. Introduction

Malignant tumors pose significant therapeutic challenges due to their heterogeneity and specific microenvironment [1,2,3,4]. Photodynamic therapy (PDT) emerges as an efficient treatment modality with several unique advantages: it is operationally simple, cost-effective, noninvasive, and does not induce drug resistance [5,6,7,8,9]. These attributes make PDT, which kills cancer cells through the generation of highly reactive oxygen species (ROS), an exceptionally promising and widely researched antitumor approach [10,11,12,13]. The process of PDT involves a light-activated photosensitizer facilitating energy transfer or electron transfer to produce oxidative ROS [14,15,16]. Among various photosensitizers, porphyrins and their derivatives, characterized by four pyrrole rings conjugated with methine bridges, stand out for their excellent biocompatibility, photostability, and singlet-oxygen-generation capability [17,18,19]. Despite these advantages, porphyrin molecules, especially those with tetraphenyl structures, exhibit strong hydrophobicity leading to aggregation in aqueous solutions [20,21,22]. Thus, developing carriers that enhance the dispersion stability and accumulation concentration of porphyrin photosensitizers at the tumor site is crucial for improving PDT efficacy [23,24].

Among the diverse carrier materials, researchers have found that designing nanoscale metal–organic frameworks (nMOFs) by coordinating porphyrins with metals significantly enhances the physiological solution dispersibility, singlet oxygen production efficiency, and accumulation capacity of porphyrin photosensitizers [25,26,27,28]. Numerous materials are used as carriers for porphyrin in phototherapy, but nMOF materials exhibit distinct advantages. Polymers can be employed to achieve the loading of porphyrin molecules; however, this method relies on the interaction between hydrophobic blocks of amphiphilic polymers and hydrophobic porphyrin molecules, which does not entirely prevent the aggregation of porphyrin molecules. Liposomes can also be utilized for loading porphyrin molecules, yet they often fail to achieve optimal loading capacity and cannot avoid the aggregation of porphyrin molecules. Inorganic materials such as silica spheres can also load porphyrin molecules, but the aggregation of porphyrin within these carriers commonly leads to quenching, affecting the production of ^1^O_2_ by porphyrin molecules. In contrast, incorporating porphyrin into framework materials to prepare nMOFs offers several advantages. Firstly, the periodic and ordered structure of nMOFs can completely prevent the aggregation of hydrophobic porphyrin molecules. Secondly, the porous nature of porphyrin-based nMOFs facilitates the diffusion of O_2_ molecules into the material and the subsequent diffusion of produced ^1^O_2_ outwards. Additionally, the porous structure of porphyrin-based nMOFs supports the loading of other drug molecules, enabling combined therapeutic effects. This makes porphyrin-based nMOFs one of the most promising candidates for clinical applications requiring photoactive materials. Lastly, the large specific surface area of porphyrin-based nMOFs facilitates further modifications with polymers to enhance their targeting capability [29,30]. Therefore, phototherapy materials based on porphyrin-based nMOFs possess significant research value. However, high intracellular glutathione (GSH) concentrations in cancer cells, aimed at maintaining redox homeostasis, can greatly limit the effectiveness of ROS-generating therapies such as PDT, sonodynamic therapy (SDT), chemodynamic therapy (CDT), ferroptosis therapy, and radiotherapy [31,32]. Reducing intracellular GSH levels to prevent ROS scavenging is therefore an effective strategy to enhance PDT efficacy [33].

Intracellular glutathione peroxidase can lower GSH levels, but the large molecular structure of glutathione peroxidase necessitates complex delivery system designs [31]. The inherent instability of enzymes may impose limitations on their prolonged application as therapeutic agents for modulating GSH levels. Interestingly, GSH, as a thiol-containing reductant, can reduce various metal ions including Cu^2+^, Mn^3+^, and Fe^3+^ [34,35,36,37,38,39]. Notably, these metal ions can coordinate with meso-tetra(4-carboxyphenyl)porphyrin (TCPP) molecules to form MOF materials [34]. This enables a straightforward combination of GSH depletion and porphyrin-mediated PDT without the need for a complex design, presenting a promising method for treating malignant tumors [36]. Moreover, under the weakly acidic conditions within cancer cells, GSH can disrupt the structure of Cu^2+^-, Mn^3+^-, and Fe^3+^-based nMOFs, releasing porphyrin photosensitizers [34]. This further promotes ^1^O_2_ production and fluorescence recovery, enabling fluorescence imaging-guided therapy [34]. Importantly, this GSH-activated enhanced PDT effect can also minimize damage to normal cells by photoactive nMOFs.

**Figure 1:**
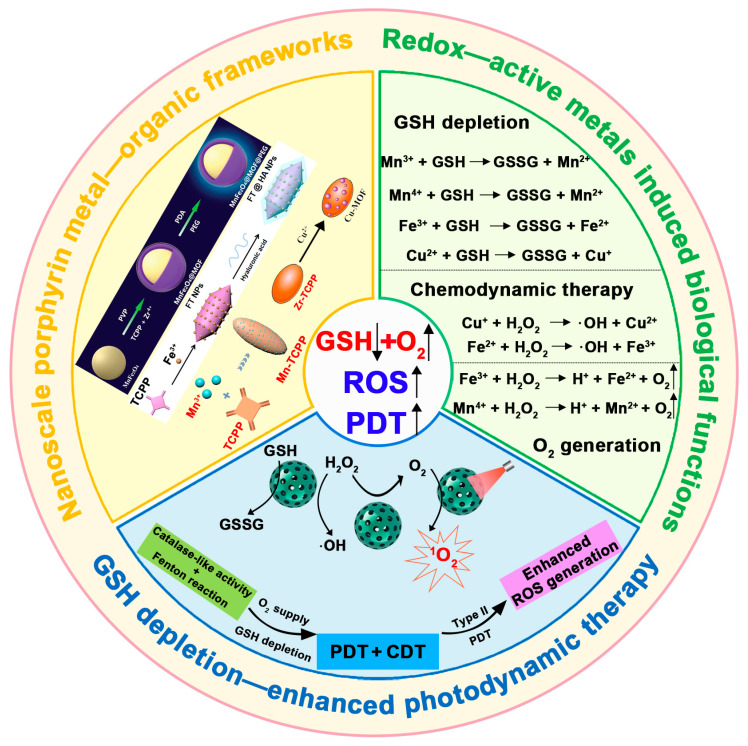
Schematic illustration of the synthesis of porphyrin-based nMOFs, the redox reaction activity of metal ions within their framework, and their biomedical functions. Additionally, this figure explains the mechanism by which nMOFs exhibit enhanced PDT efficacy following GSH depletion. Reproduced with permission from [34,35,36,37]. Copyright (2019), American Chemical Society (Washington, DC, USA). Copyright (2019), John Wiley & Sons, Ltd. (Hoboken, NJ, USA). Copyright (2020), American Chemical Society (Washington, DC, USA). Copyright (2023), The Royal Society of Chemistry (Cambridge, UK).

In 2021, Yang et al. systematically and comprehensively discussed the progress of engineered nanomedicines in enhancing cancer therapies through GSH depletion [31]. Wu et al. summarized the synergistic therapeutic effects of GSH-scavenging nanomedicines in conjunction with PDT, CDT, radiotherapy, and chemotherapy [32]. Niu et al. further elucidated how the GSH depletion mechanism enhances treatments based on ROS, promotes ferroptosis, and improves chemotherapy efficacy [33]. Subsequently, Wang et al. specifically highlighted the enhancement effects of GSH scavenging on SDT [40]. This review concentrates on an in-depth summary of recent research over the past few years concerning the enhancement of PDT efficacy using porphyrin-based nMOF materials through GSH-scavenging capabilities. It details the molecular mechanisms of various systems designed to reduce GSH, and compares the impact of GSH presence on the ^1^O_2_ yield from porphyrin-based nMOFs under light exposure. This review systematically outlines the methodologies for integrating redox-active metals within porphyrin-based nMOFs, assessing both the strengths and challenges associated with each approach (Figure 1). It also elucidates the advantages of employing redox-active metals as formulations for GSH depletion. These metals offer several key benefits: (1) they can directly and effectively deplete GSH via high-valence states [34]; (2) they can also mimic catalase activity to catalyze the decomposition of the higher concentrations of H_2_O_2_ found in cancer cells into O_2_, which not only mitigates hypoxia in cancerous tissues but also facilitates the generation of ^1^O_2_ [35]; (3) upon reduction, they can react with the high concentrations of H_2_O_2_ within cancer cells to yield hydroxyl radicals—potent oxidants that markedly augment the therapeutic potential of ROS-based treatments (Figure 1) [36]. Finally, this review evaluates the therapeutic effects and application prospects of these systems on malignant tumors and infected wounds at both cellular and animal levels. The review aims to provide valuable insights for the clinical research of porphyrin-based nMOFs.

## 2. Advantages of Metals as GSH Depleters and Effects of Loading Methods

**Figure 2:**
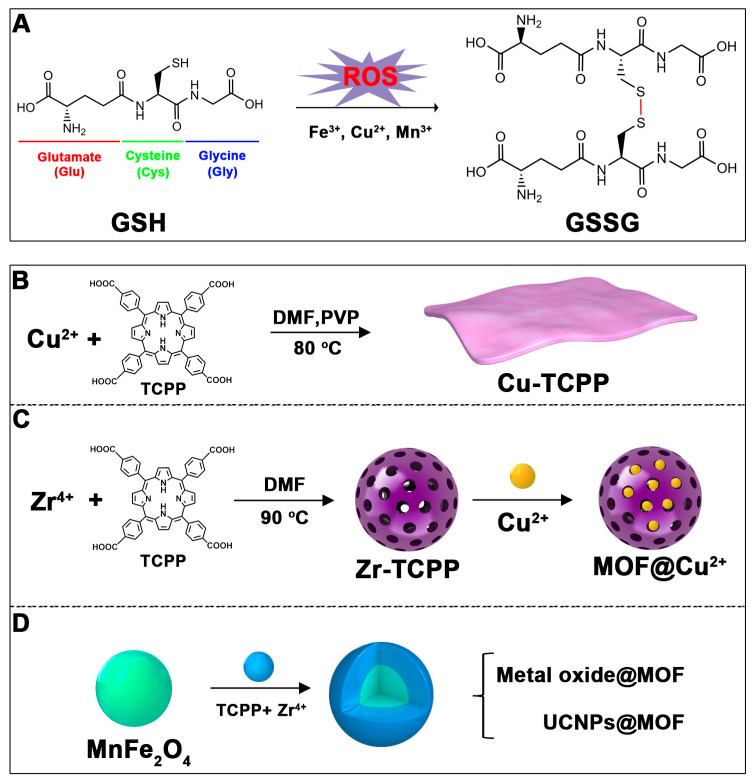
(**A**) The molecular structure of GSH and its reaction with ROS or metal ions to form GSSG. (**B**) Schematic illustration of Cu^2+^ coordination with TCPP to form the Cu-TCPP MOF. (**C**) Illustration of Zr^4+^ coordination with TCPP molecules to form the Zr-TCPP MOF, and its subsequent loading with Cu^2+^ ions. (**D**) Schematic representation of Zr-TCPP MOF growth on the surface of inorganic oxides or UCNPs.

Cancer cells have evolved various reductive mechanisms to counteract the effects of ROS on their survival [41]. These reductive substances include GSH, vitamin C, vitamin E, thioredoxin reductase, catalase, superoxide dismutase (SOD), and others [41]. Among them, GSH—a tripeptide composed of glutamic acid (Glu), cysteine (Cys), and glycine (Gly)—has been extensively studied due to its unique role within cells (Figure 2A) [31]. GSH is one of the most important antioxidants in cancer cells, capable of neutralizing ROS by forming its oxidized form, glutathione disulfide (GSSG), thereby mitigating the therapeutic impact of ROS-based treatments (Figure 2A) [32]. Reducing intracellular GSH levels not only amplifies the efficacy of ROS-mediated therapies but also diminishes the resistance of some cancer cells to chemotherapeutic agents, presenting a novel approach for treating drug-resistant cancer cells [32]. Strategies to decrease GSH concentration in cancer cells are numerous, including the pretreatment of phenethyl isothiocyanate, quercetin, buthionine sulfoximine (BSO), and valproic acid (VPA), among others. These molecules reduce cellular GSH either by directly scavenging GSH or inhibiting its synthesis pathways [42]. However, these compounds often require structural modifications to enhance their therapeutic potency. Moreover, they typically only serve to lower GSH levels without providing additional biomedical functionalities.

Research has shown that certain redox-active metals can not only interact directly with GSH to reduce its concentration but also react with H_2_O_2_ to produce O_2_, thus alleviating hypoxic microenvironments that limit PDT [34]. Additionally, these processes can generate lower-valent metals which then react with intracellular H_2_O_2_ to produce highly oxidative hydroxyl radicals, further enhancing ROS-mediated therapeutic effects [36]. Another advantage of these metals is their stability and ability to restore their oxidation state through reactions with H_2_O_2_, allowing repeated GSH depletion. Furthermore, some of these metals possess CT or MR imaging capabilities, potentially integrating diagnostics with therapy [34]. Upon reviewing the literature, it was found that porphyrin-based nMOFs can incorporate metals through different methods. The first method involves coordination with TCPP as linker to form the MOF structure (Figure 2B). This loading method is relatively simple and allows for a high metal load. The second method involves synthesizing linear porphyrin-based MOFs and subsequently coordinating divalent ions such as Cu^2+^ at defect sites of the carboxylate ligands to achieve functional metal loading (Figure 2C). This method facilitates interaction between the loaded metals and intracellular GSH, although it may result in lower metal loads. The third method entails constructing core–shell structures or other composites using porphyrin-based nMOFs combined with metal oxides or upconversion nanoparticles (UCNPs) (Figure 2D). This approach offers advantages, such as introducing new functionalities from metal oxides or upconversion materials, although the preparation process is more complex and the reaction of metal oxides with intracellular GSH might be hindered if used as the core structure. Overall, porphyrin-based nMOFs provide multiple pathways for achieving effective GSH depletion when combined with metals, enabling the delivery of various metals and their oxides into cells while exerting corresponding nanomedicine functions. Therefore, metal-induced GSH depletion to enhance the PDT efficacy of porphyrin-based nMOFs is not only effective and feasible but also holds significant application potential.

## 3. Synthesis of Porphyrin-Based nMOFs and Their GSH Depletion Mechanisms

MOFs have garnered extensive attention as biomedicine carrier materials due to their diversity and designability [43,44]. Numerous studies and developments have focused on designing systems that reduce intracellular GSH levels, thereby enhancing the inhibitory effects of MOF materials on cancer cells [45,46]. Notably, GSH depletion can significantly augment the anticancer efficacy of porphyrin-based nMOFs (Table 1). For instance, Cai et al. fabricated an Fe-coordinated porphyrin ligand molecule and coordinated it with Cu^2+^ ions to obtain Cu-TCPP(Fe) MOF materials (Table 1) [45]. They then utilized a nucleation method to grow Au NPs with an average diameter of 2.8 nm on the MOF surface and loaded RSL3 (an inhibitor of glutathione peroxidase 4 (GPX4)) molecules via π-π stacking. The authors found that Au NPs exhibit glucose oxidase-like activity, leading to substantial consumption of intracellular glucose and hindering GSH synthesis pathways. Moreover, the presence of Cu^2+^ ions can oxidize GSH into its oxidized form, GSSG, effectively depleting cellular reductive substances and enhancing ferroptosis damage (Table 1). Jiang et al. reported that Cu-TCPP can overcome dependencies on oxygen and external excitation light sources (Table 1) [46]. It selectively kills tumor cells by utilizing excess hydrogen peroxide within cancer cells. In the weakly acidic environment inside cancer cells, TCPP molecules in the Cu-TCPP nanoplatform can be peroxidized by excessive hydrogen peroxide through the Russell mechanism. In the presence of Cu-TCPP and trace amounts of Cu^2+^ ions, this leads to the generation of peroxyl radicals (ROO·), which subsequently undergo rearrangement reactions within cells to produce singlet oxygen. The generated Cu^2+^ ions lower intracellular GSH concentration, thus preventing these antioxidants from protecting cells against oxidative damage. Ultimately, this system achieves enhanced chemotherapy by depleting GSH, exploiting the microenvironment of hypoxic tumors for effective inhibition (Table 1). Zhao et al. used disulfide-linked imidazole ligands coordinated with Zn^2+^ ions to prepare MOF NPs and loaded them with Chlorin e6 (Ce6) molecules to obtain Ce6@RMOF materials [47]. The presence of disulfide bonds enables the oxidation of GSH into GSSG. The depletion of GSH further leads to the inactivation of GPX4, ultimately achieving the combined therapeutic effects of ferroptosis and PDT (Table 1). Beyond metal ions with variable valence states and disulfide/thiol transformations, azo bonds may also lead to the reduction of intracellular GSH and other reductants [48]. For example, Guo et al. developed an MOF using azo-containing imidazole ligands coordinated with Zn^2+^ ions as a carrier for the photosensitizer Ce6, forming the MOF@Ce6 nanoplatform [48]. Within hypoxic cancer cells, azoreductases degrade the MOF@Ce6, simultaneously depleting nicotinamide adenine dinucleotide phosphate (NADPH). Since NADPH serves as an electron donor for reductive substances including GSH and thioredoxin (Trx), its reduced concentration decreases intracellular GSH and Trx levels, amplifying the PDT effect produced by Ce6-generated ROS (Table 1). Zhang et al. prepared a nanoscale copper-coordinated polymer (CCP) by coordinating Cu^2+^ with Zn protoporphyrin IX [49]. Intracellular GSH reacts with Cu^2+^ within the CCP framework, generating Cu^+^ ions. This process not only lowers intracellular GSH concentrations but also releases Zn protoporphyrin IX, which effectively inhibits the activity of the antioxidant heme oxygenase-1. Furthermore, the generated Cu^+^ ions react with intracellular hydrogen peroxide to produce hydroxyl radicals, undergoing a chemodynamic process, ultimately enhancing the CDT anticancer effect by inhibiting intracellular reductive substances (Table 1). Yang et al. formed the highly chemically stable PCN-224 MOF material by coordinating Zr^4+^ with TCPP and achieved Cu^2+^ loading through PCN-224’s unique structure, obtaining the PCN-224(Cu) nanoplatform [50]. Cu^2+^ in this nanoplatform reduces intracellular GSH concentrations, while the generated Cu^+^ undergoes the Fenton reaction with H_2_O_2_ to produce hydroxyl radicals, resulting in an enhanced ROS-mediated suppression of cancer cell proliferation (Table 1). Lin et al. demonstrated that ZIF-8 has oxygen storage capacity, though weaker than Cu^2+^-based MOFs [51]. Therefore, they doped Cu^2+^ into the ZIF-8 framework to enhance O_2_ storage capability and integrated Ce6 photosensitizer molecules into the nanoplatform. Upon entering cells, the platform releases Cu^2+^, which reduces GSH concentration, and the generated Cu^+^ ions react with hydrogen peroxide to produce hydroxyl radicals for CDT. Finally, this nanoplatform realizes synergistic CDT and PDT effects enhanced by Cu^2+^-induced oxygen storage and GSH depletion (Table 1). Xu et al. prepared Mn-coordinated TCPP molecules (Mn-TCPP) and synthesized Mn-MOF via solvothermal reactions with Zr^4+^ ions [52]. They discovered that the Mn-MOF nanoplatform reacts with excess H_2_O_2_ within cancer cells, producing oxygen through Mn^3+^/Mn^4+^ dynamic conversion to improve the hypoxic state. Meanwhile, the metal nodes of the Mn-MOF lower intracellular GSH concentration, realizing enhanced SDT and ferroptosis combined treatment mechanisms (Table 1).

These systems demonstrate that reducing intracellular GSH in cancer cells is an effective and worthwhile strategy for antitumor proliferation, especially when combined with treatments such as CDT, SDT, ferroptosis, and PDT, all of which rely on ROS to inhibit cancer cell proliferation. The modifiability and multifunctionality of MOFs allow these combined therapies to be seamlessly integrated into a single nanoplatform. This review mainly summarizes the role mechanisms and antitumor principles of research work involving metal ions (Mn^3+^, Mn^4+^, Fe^3+^, and Cu^2+^) that consume intracellular GSH to enhance the PDT-related treatment effects of MOF nanoplatforms based on TCPP molecules (Table 2). Summarizing these research efforts provides new insights into how porphyrin-based nMOFs can exploit the unique tumor microenvironment of cancer cells to achieve enhanced therapeutic effects.

## 4. Mn(III)-TCPP nMOF-Induced GSH Depletion for Enhanced PDT

Porphyrin photosensitizers exhibit excellent biocompatibility, but their poor water solubility has limited their application in biomedical fields [53]. Extensive research indicates that constructing porous MOFs through the coordination of porphyrin molecules can significantly enhance the delivery efficacy of porphyrin photosensitizers to cancer cells [5]. However, traditional metal–porphyrin MOFs directly generate ROS upon light exposure, which cannot selectively target tumor tissues and may affect normal tissues. Therefore, developing a photo-inert porphyrin-based MOF formulation that only becomes phototherapeutically active in response to the tumor microenvironment is an essential area of study [34].

**Figure 3:**
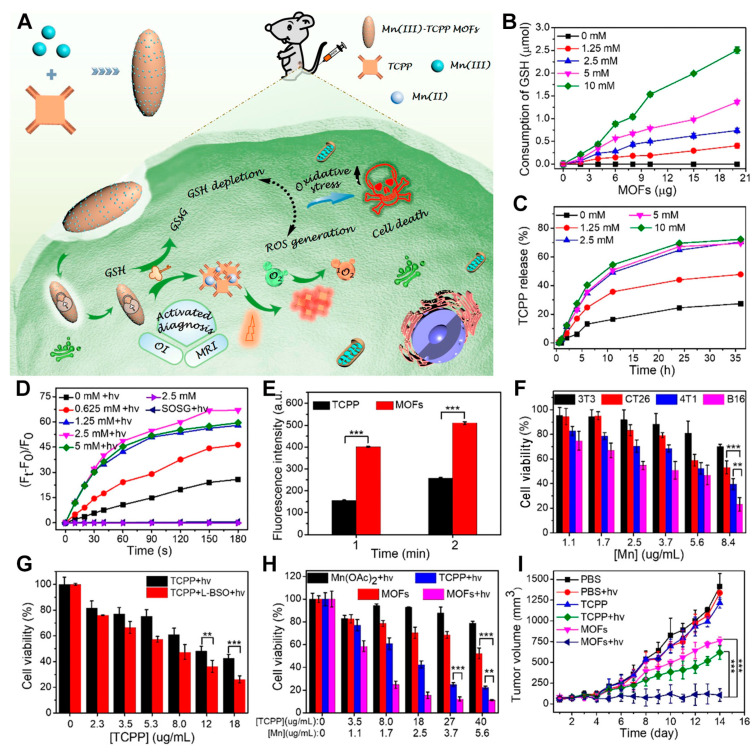
(**A**) Schematic illustration of the preparation of Mn(III)-TCPP nMOFs and their in vivo GSH-responsive depletion, followed by activation of fluorescence imaging and MRI-guided enhanced PDT therapy. (**B**) The effect of varying amounts of MOF material on the consumption of GSH at different concentrations. (**C**) The effect of different GSH concentrations on the release of TCPP molecules from MOFs. (**D**) ROS-generation ability detected by SOSG. (**E**) TCPP and MOF-induced ROS-generation ability (10 mM GSH). *** *p* < 0.001. (**F**) The cell viability of Mn-TCPP MOFs against four cell lines. (**G**) Cell viability under different incubation conditions (TCPP + hv, TCPP + L-BSO + hv). ** *p* < 0.01, *** *p* < 0.001. (**H**) Cell viability after treatment with four incubation conditions. ** *p* < 0.01, *** *p* < 0.001. (**I**) Tumor volume changes from different groups after 14 days. *** *p* < 0.001. Reproduced with permission from [34]. Copyright (2019), American Chemical Society.

For instance, Zhang et al. employed a one-pot synthesis method using Mn^3+^ ions and TCPP as nodes and linker molecules to fabricate Mn-TCPP materials (Figure 3A) [34]. The resultant Mn-TCPP exhibits inert behavior; it remains quenched in fluorescence and incapable of ROS generation within normal tissue cells. Upon endocytosis by cancer cells, however, the Mn-TCPP interacts with the excess GSH inside these cells. The Mn^3+^ ions are reduced to Mn^2+^ ions, leading to the release of TCPP molecules into a free state. Under light irradiation, the released TCPP generates ^1^O_2_ that targets intracellular biomolecules, while GSH depletion facilitates ROS accumulation within the cell, thereby enhancing the PDT effect. Subsequent experiments revealed that the consumption of GSH increased significantly with higher concentrations of MOFs (Figure 3B), and that the release of TCPP correspondingly increased with elevated GSH levels (Figure 3C). The singlet oxygen sensing group (SOSG) assay confirmed that Mn-TCPP could be structurally disrupted by GSH, releasing TCPP and enabling the PDT process (Figure 3D). As shown in Figure 3D, the capacity for ROS production under light increased with rising GSH concentration up to 2.5 mM. Beyond this point, further increases in GSH concentration led to decreased ROS levels due to GSH’s own ROS-scavenging ability. Considering the high concentration of GSH (up to 10 mM) in tumor cells, the authors tested the singlet-oxygen-generating capability of TCPP and Mn-TCPP in a 10 mM GSH solution. They found that Mn-TCPP generated twice as much singlet oxygen as free TCPP molecules under identical conditions (Figure 3E). This result not only demonstrates the enhanced PDT mechanism of the Mn-TCPP system in response to intracellular GSH but also shows its effectiveness even at high GSH concentrations. The authors then evaluated the cytotoxicity of the material on different cell lines with varying GSH concentrations. The in vitro MTT assay, a commonly used method for evaluating the cytotoxicity of nanomedicines, was adopted by the authors to determine the therapeutic mechanism of Mn-TCPP under various conditions [54,55,56,57,58]. As depicted in Figure 3F, compared with the normal 3T3 cell line, the cancer cell lines CT26, 4T1, and B16 showed increased cytotoxicity due to their relatively higher GSH levels. Additionally, the inclusion of a GSH synthesis inhibitor (L-buthionine sulfoximine, L-BSO) in cell cultures enhanced PDT efficacy as expected (Figure 3G). Cytotoxicity assays (Figure 3H) revealed that the MOFs plus light treatment group exhibited the lowest cell viability, demonstrating the strongest inhibition of cancer cell proliferation. Furthermore, Figure 3I illustrates that MOFs achieved optimal suppression of 4T1 cancer cell volume under 660 nm laser irradiation. Employing the Mn^3+^ ion encapsulation strategy, this system achieves the controlled release of TCPP photosensitizer molecules in response to the GSH-rich microenvironment and enhances PDT efficacy by reducing GSH levels. It serves as a reference for developing safer and more reliable porphyrin-based MOF systems for phototherapy research, providing insights into improving the selective targeting of tumors while minimizing damage to healthy tissues. However, it should be noted that although the GSH depletion capability of nanomaterials can effectively enhance the efficacy of PDT, the hypoxic environment of solid tumor cells may still lead to a lower production of singlet oxygen, thereby resulting in suboptimal PDT effectiveness.

## 5. MnFe_2_O_4_-Induced GSH Depletion for Enhanced PDT

**Figure 4:**
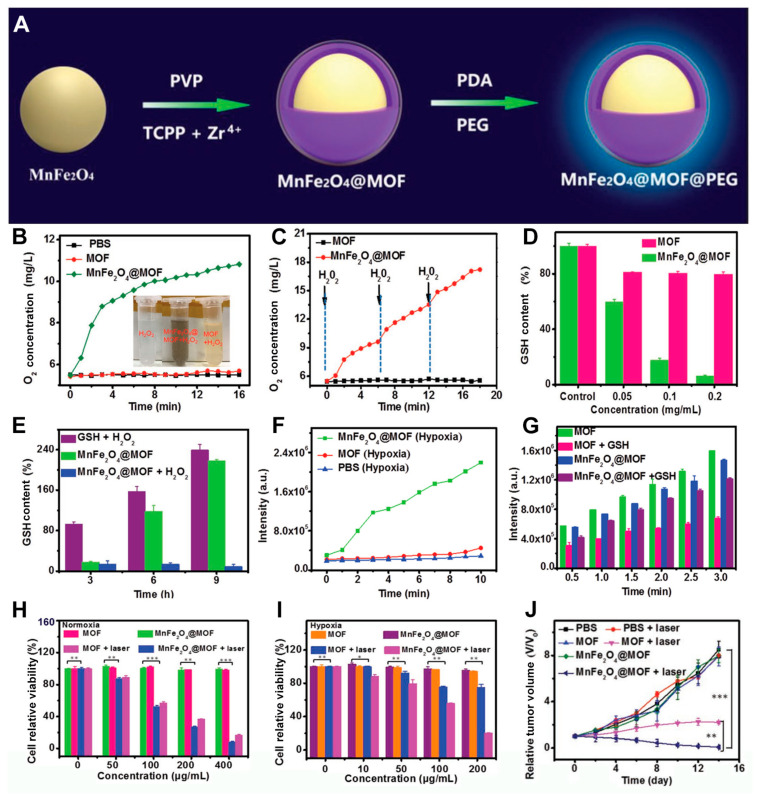
(**A**) Synthesis procedure of MnFe_2_O_4_@MOF@PEG composite material. (**B**) Comparison of O_2_ generation by MOF and MnFe_2_O_4_@MOF materials after H_2_O_2_ addition. (**C**) Comparison of O_2_ generation by MOF and MnFe_2_O_4_@MOF materials after three additions of H_2_O_2_. (**D**) Comparison of GSH consumption by different concentrations of MOF and MnFe_2_O_4_@MOF materials. (**E**) Variations in GSH levels under three different conditions. (**F**) Comparison of ^1^O_2_ generation by MOF and MnFe_2_O_4_@MOF materials under hypoxic conditions. (**G**) Comparison of ^1^O_2_-generation capacity after treatment under four different conditions (using SOSG as a ^1^O_2_ scavenger). (**H**) Comparison of cell viability following treatment under four distinct conditions in normoxic conditions. (** *p* < 0.01 and *** *p* < 0.001). (**I**) Comparison of cell viability following treatment under four distinct conditions in hypoxia conditions (* *p* < 0.05, ** *p* < 0.01). (**J**) Changes in relative tumor volume in mice under six different conditions (** *p* < 0.01 and *** *p* < 0.001). Reproduced with permission from [35]. Copyright (2019), John Wiley & Sons, Ltd.

Because cancers are known for their high lethality, the field of cancer treatment research has already consumed a considerable amount of researchers’ manpower and time investment [59,60,61,62,63,64,65,66,67,68]. Researchers have found that cancer cells exhibit unique characteristics such as hypoxic environments and elevated levels of reductive substances such as GSH, which significantly limit the efficacy of PDT, a process that relies on oxygen for its therapeutic action [31,32,33]. To address this hypoxic microenvironment, researchers have adopted various strategies, such as using hemoglobin or perfluorocarbons to carry oxygen [69,70], inhibiting mitochondrial respiration to directly cut off cellular oxygen consumption pathways [71,72,73,74,75,76], employing catalase to produce O_2_ from H_2_O_2_ [77,78,79,80,81,82,83], and utilizing Pt NPs or MnO_2_ nanotechnology to catalyze O_2_ generation from H_2_O_2_ [84,85,86,87,88]. These studies underscore the importance of improving oxygen concentration for PDT. Research has also indicated that inorganic NPs such as Mn^4+^ or Fe^3+^ can effectively catalyze oxygen production from H_2_O_2_ through a cyclic process. This Fenton-like reaction not only generates oxygen but also oxidizes GSH into GSSG in the presence of hydrogen peroxide, serving as a critical step to prevent reduced PDT efficacy. Moreover, these metal-based oxides are easily surface-modified and can form core–shell structures, facilitating the preparation of composite materials combining inorganic oxides with porphyrin-based nMOFs. For example, Zhang et al. initially prepared polyvinylpyrrolidone (PVP)-coated MnFe_2_O_4_ inorganic oxide particles, which were then used to adsorb Zr^4+^ ions and tetracarboxylic acid porphyrins, resulting in the MnFe_2_O_4_@MOF nanocomposite (Figure 4A) [35]. This composite material enhances ROS concentration by concurrently increasing O_2_ levels and depleting GSH, thereby potentiating PDT efficacy (Figure 4A). Specifically, the authors first confirmed the O_2_-generating function of the material in the presence of H_2_O_2_. As shown in Figure 4B, MnFe_2_O_4_@MOF efficiently produced O_2_ when H_2_O_2_ was present, whereas standalone Zr-TCPP MOF did not catalyze O_2_ generation from H_2_O_2_. The peroxidase-like activity of MnFe_2_O_4_ exhibited sustained effects, repeatedly catalyzing new additions of H_2_O_2_ into O_2_ (Figure 4C). Subsequently, the authors verified the glutathione peroxidase-like activity of the prepared composite. As depicted in Figure 4D, compared with the MOF treatment group, MnFe_2_O_4_@MOF significantly reduced GSH concentration, a process requiring H_2_O_2_ participation (Figure 4E). Importantly, experiments revealed that even under hypoxic conditions, MnFe_2_O_4_@MOF could effectively generate ^1^O_2_ upon light irradiation due to its intrinsic capability to catalyze O_2_ production from H_2_O_2_ (Figure 4F), offering new opportunities for treating hypoxic tumors. Furthermore, it was found that more than fifty percent of the ^1^O_2_ generated by MOF alone was consumed by GSH post-irradiation (Figure 4G). In contrast, less than 20% of the ^1^O_2_ produced by MnFe_2_O_4_@MOF was consumed by GSH, owing to the composite’s autonomous GSH depletion function, which indeed contributed to increased intracellular ROS concentration (Figure 4G). The authors demonstrated that MnFe_2_O_4_@MOF effectively reduced cell viability under both normoxic and hypoxic conditions when irradiated with 660 nm laser light (Figure 4H,I). In vivo studies on mouse breast cancer 4T1 models showed that MnFe_2_O_4_@MOF significantly decreased tumor volume under light irradiation, indicating promising therapeutic outcomes (Figure 4J). This system successfully achieved autonomous O_2_ generation and effective GSH reduction, enhancing PDT treatment efficacy. It offers new insights for therapies dependent on oxygen or GSH depletion, providing valuable references for the future biomedical applications and precise treatments of porphyrin-based nMOFs. This work presents an efficient approach to overcoming the limitations posed by the tumor microenvironment, significantly advancing the field of PDT. However, the system lacks active targeting functionality after administration, limiting its accumulation at the tumor site.

## 6. Fe-TCPP nMOF-Induced GSH Depletion for Enhanced PDT

**Figure 5:**
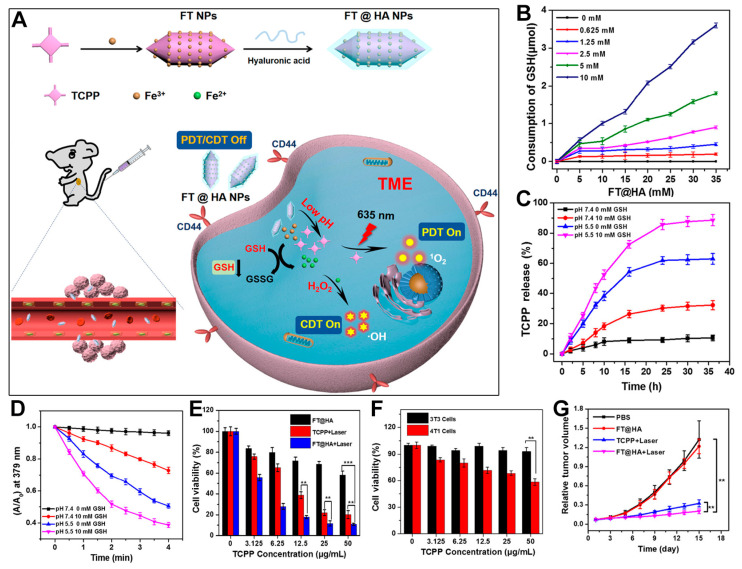
(**A**) Schematic illustration of the preparation of FT@HA NPs and their in vivo synergistic antitumor mechanism. (**B**) The effect of different concentrations of FT@HA NPs on the consumption of GSH at varying concentrations. (**C**) TCPP release from FT@HA NPs triggered by pH and GSH. (**D**) ^1^O_2_-generation capability of FT@HA NPs under different pH and GSH concentration conditions (with ABDA as a ^1^O_2_ scavenger). (**E**) Comparison of the viability of 4T1 cells following treatment under three distinct conditions. (**F**) Assessment of cell viability in 4T1 and NIH-3T3 cells after exposure to FT@HA NPs. ** *p* < 0.01, *** *p* < 0.001. (**G**) Tumor volume changes after different treatments. Reproduced with permission from [36]. Copyright (2020), American Chemical Society.

To enhance the targeting efficacy of porphyrin-based MOFs, surface modification is critical. Researchers have found that employing a biomimetic strategy by coating the MOF materials with cell membranes can effectively improve their targeting specificity towards cancer cells [36]. Additionally, encapsulating the NPs with hyaluronic acid (HA) can promote targeting to cancer cells that overexpress CD44 receptors on their surface [89,90,91,92]. For instance, Fan et al. first synthesized Fe-TCPP MOF NPs (FT NPs) and then utilized electrostatic interactions to modify their surface with HA polymer, resulting in FT@HA NPs (Figure 5A) [36]. The authors demonstrated that FT@HA NPs could be efficiently internalized by cells, achieving combined CDT initiated by Fe^3+^ ions and enhanced PDT through GSH depletion. As shown in Figure 5B, the consumption of GSH increased with higher concentrations of FT@HA NPs and GSH. The authors verified that FT@HA NPs exhibited dual responsiveness to pH and GSH levels. At pH 5.5 and 10 mM GSH, the structural degradation of FT NPs was significantly enhanced, leading to the release of TCPP photosensitizer molecules (Figure 5C). This unique responsiveness to the intracellular environment of cancer cells minimizes damage to normal cells. Experimental results indicated that after treatment under conditions of pH 5.5 and 10 mM GSH, the singlet-oxygen-generation capacity of FT@HA NPs, as measured by ABDA assay, was notably increased (Figure 5D). The authors further evaluated the in vitro antitumor effects of the materials. As depicted in Figure 5E, due to their weak acidity and GSH responsiveness, FT@NPs exhibited mild cytotoxicity via Fenton reaction. Under light irradiation, the TCPP photosensitizer showed certain cytotoxicity. However, the FT@NPs treatment group demonstrated the lowest cell viability when activated by GSH and weak acidity. Moreover, the cellular uptake efficiency of different cells was examined. As shown in Figure 5F, since 4T1 cells overexpress CD44 receptors on their surface, they exhibited significantly higher uptake of FT@HA NPs compared with cells without overexpression of CD44 protein receptors. This indicates that surface modification with HA polymer enhances the active targeting effect of the material towards cancer cells. Consistent with the cytotoxicity results, the inhibition of 4T1 tumors in mice (Figure 5G) revealed that FT@HA NPs, when activated by intracellular weak acidity and GSH, exhibited effective suppression of tumor growth through the synergistic effects of PDT and CDT. This system provides new insights into the design of targeted therapies for porphyrin-based MOFs and microenvironment-responsive PDT strategies tailored to the pH and GSH characteristics of cancer cells. This work highlights the importance of surface modifications for enhancing the targeting capabilities of porphyrin-based MOFs. By leveraging the dual pH and GSH responsiveness, along with the active targeting properties provided by HA, the developed nanomaterials achieve improved therapeutic outcomes while minimizing off-target effects. This approach paves the way for more precise and effective cancer treatments. In this system, the author investigated the hydroxyl radicals generated by the Fenton-like system of Fe^3+^/H_2_O_2_. However, studies have shown that Fe^3+^ disappears and forms Fe(OH)_3_ precipitates when the pH is greater than 4. Therefore, strict acidic conditions (pH < 4) may be required for practical application. In contrast, the Cu^2+^/H_2_O_2_ Fenton-like system can operate over a broader pH range [93]. Therefore, investigating Fenton-like reactions based on Cu elements may lead to unexpected enhancements in the phototherapy effects of porphyrin-based nMOFs.

## 7. Cu-MOF-Induced GSH Depletion for Enhanced PDT

**Figure 6:**
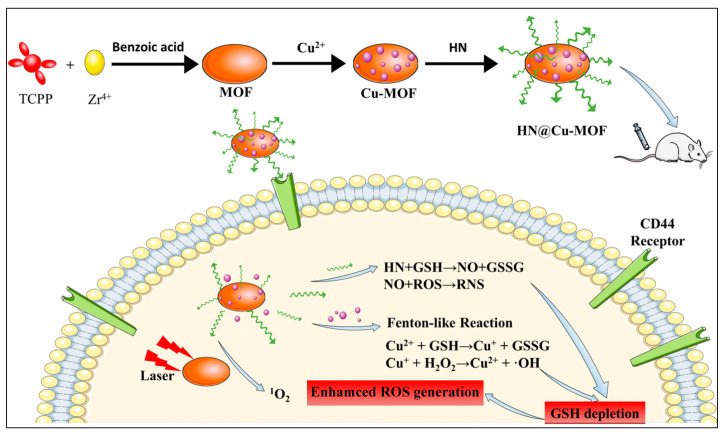
An overview of the fabrication procedure for HN@Cu-MOF and a schematic depiction of its therapeutic functions in PDT, CDT, and NO gas therapy. Reproduced with permission from [37]. Copyright (2023), The Royal Society of Chemistry.

In recent years, green gas therapy has emerged as a widely researched novel therapeutic approach [94,95,96,97]. This treatment modality primarily includes gases such as carbon monoxide (CO), nitric oxide (NO), sulfur dioxide (SO_2_), and hydrogen sulfide (H_2_S) [98,99,100]. Among these, NO, acting as a free radical, exhibits dual functionalities: at low concentrations, it can prevent cell apoptosis and promote cancer cell metastasis; however, at higher concentrations, NO induces cell apoptosis through cytotoxic reactions or cellular organelle dysfunction, thereby demonstrating antitumor activity [10]. Moreover, the NO gas could stimulate vasodilation which aids in restoring O_2_ concentration and enhancing the efficacy of PDT [37]. The integration of NO therapy with PDT has become a focal point of extensive research [100]. Additionally, studies have shown that under weakly acidic conditions, the reaction rate between Cu^2+^/Cu^+^ significantly increases compared with the transformation rate between Fe^3+^/Fe^2+^ [37]. Therefore, Cu-based Fenton reactions offer certain advantages over Fe-based systems. For example, Yuan et al. utilized Zr^4+^ ions coordinated with TCPP to prepare MOF materials [37]. Following this, they employed a straightforward ion-doping strategy to obtain Cu-MOF materials. They then conjugated NO donor-modified HA with the MOFs via electrostatic interactions, resulting in HN@Cu-MOF composite materials (Figure 6). HA facilitates the accumulation of nanomaterials in specific cancer cells rich in CD44 surface glycoproteins. Once inside the cells, the composite materials interact with intracellular GSH, not only depleting GSH levels but also releasing NO gas. Nitric oxide can react with ROS generated by light-activated materials to produce reactive nitrogen species (RNS). Through doping technology, the Cu^2+^ ions within the composite material can interact with intracellular GSH to produce Cu^+^ ions. Subsequently, the generated Cu^+^ can react with intracellular H_2_O_2_, utilizing the Fenton-like reaction mechanism to produce highly oxidative hydroxyl radicals (Figure 6). This system effectively combines GSH depletion-enhanced NO gas therapy, PDT, and CDT for a potent inhibition of cancer cells. The simplicity and effectiveness of this system provide valuable insights into the application of porphyrin-based MOF materials. This work highlights the development of a multifunctional therapeutic platform that integrates NO release, ROS generation, and Cu^+^-mediated Fenton reactions. By leveraging the unique properties of Cu-based Fenton reactions and NO’s dual role in promoting and inhibiting cancer progression, this study offers a promising strategy for enhancing the therapeutic efficacy of cancer treatments. It also provides a reference for future biomedical applications of porphyrin-based MOFs, emphasizing the importance of combining multiple therapeutic modalities to target the complex nature of cancer cells. In this system, the author investigated the enhancement of PDT effects of porphyrin-based nMOFs through the reaction of GSH-induced Cu^+^ ions from Cu^2+^ with H_2_O_2_ in cancer cells to generate hydroxyl radicals. However, attention must be paid to the fact that Cu^+^ ions are extremely susceptible to oxidation by O_2_ into Cu^2+^, thereby inhibiting the Fenton-like reaction between Cu^+^ and H_2_O_2_ under slightly acidic conditions [93].

## 8. Fe(III)-Induced GSH Depletion for Enhanced PDT

**Figure 7:**
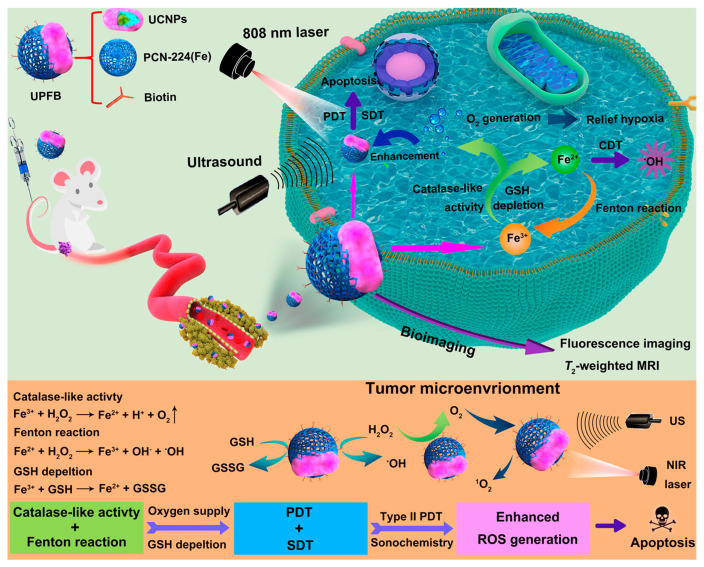
Schematic illustration of the composition of UPFB NPs and their antitumor mechanisms activated by 808 nm laser irradiation and ultrasound. Reproduced with permission from [38]. Copyright (2021), American Chemical Society.

PDT relies on the synergistic effects of photosensitizers, light sources, and oxygen [53,101]. While porphyrin-based MOFs significantly enhance the aqueous dispersion stability and tumor accumulation of hydrophobic photosensitizer molecules, their excitation wavelengths typically fall between 640 and 660 nm, limiting their effectiveness to superficial lesions and hindering penetration into deeper tissues. Consequently, for deep-seated tumors, the therapeutic efficacy of PDT may be suboptimal. Therefore, integrating two-photon or upconversion technologies with porphyrin-based photodynamic materials holds significant promise [38]. Additionally, studies have shown that not only laser irradiation but also ultrasound can activate photosensitive materials to effectively generate ROS [102,103,104,105,106,107]. Based on this premise, Wang et al. employed a ligand exchange method to obtain PVP-coated UCNPs, followed by a one-pot synthesis at 90 °C for 5 h to develop a core–shell structured composite material combining UCNPs with porphyrin-based MOFs, termed UPF (Figure 7) [38]. They further modified the surface of this composite using biotin via amide bond chemistry, resulting in multifunctional UPFB composites. Biotin enhanced the targeting capability of the material towards HeLa cells and U14 tumor cells. After cellular internalization, an 808 nm upconversion mechanism was used to convert low-energy light into higher-energy UV–visible light, which then activated the porphyrin photosensitizer to produce ROS. However, the authors noted that the energy transfer efficiency between the upconversion material and the porphyrin-based MOF in the UPF structure was relatively low. To address this issue, they incorporated SDT activation, utilizing ultrasound as an additional means to excite the photosensitive material. Furthermore, within the cell, Fe^3+^ ions from Fe-MOF could undergo redox reactions with hydrogen peroxide, generating Fe^2+^ ions and oxygen. The production of oxygen enhances ROS concentration during the PDT process (Figure 7). Moreover, Fe^3+^ can react with the abundant reductive substance GSH within cancer cells, depleting GSH levels while generating Fe^2+^ (Figure 7). This depletion facilitates the sustained presence and generation of high-concentration ROS, leading to cytotoxicity. In the weakly acidic intracellular environment, the generated Fe^2+^ can further react with H_2_O_2_ to form Fe^3+^ and highly oxidative hydroxyl radicals, which are more potent than ^1^O_2_. Thus, this system leverages 808 nm near-infrared light and ultrasound as coactivating energy sources, utilizing the catalase-like activity of Fe elements to increase O_2_ concentration, intensify GSH depletion, and induce highly oxidative hydroxyl radicals through CDT. It achieves a combined therapeutic approach of PDT, CDT, and SDT, successfully inhibiting the proliferation of U14 tumor cells (Figure 7). This system provides new insights into enhancing PDT efficacy of porphyrin-based MOFs through GSH depletion, paving the way for advanced cancer treatments.

## 9. MOF@Cu^2+^-Induced GSH Depletion for Enhanced PDT

**Figure 8:**
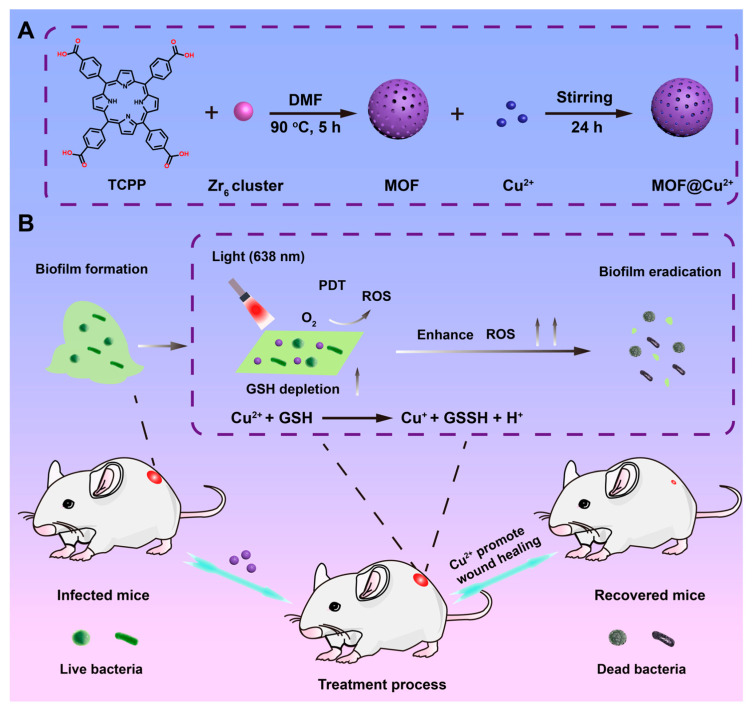
(**A**) Schematic illustration of the synthesis procedure for MOF@Cu^2+^. (**B**) Schematic illustration of how released Cu^2+^ enhances PDT antibacterial activity of MOF materials under 638 nm light by depleting GSH in bacterial biofilms. Reproduced with permission from [39]. Copyright (2024), The Royal Society of Chemistry.

GSH-enhanced PDT not only boosts the efficacy of porphyrin-based MOFs for solid tumor treatment but also holds significant promise for combating bacterial biofilm infections [39]. Bacterial infections are a major cause of mortality each year, posing a substantial threat to human health [39]. Bacteria protect themselves by secreting a polysaccharide matrix that shields them from external threats such as antibiotic treatments [39]. Recent studies have demonstrated that PDT, a noninvasive and non-resistance-inducing therapy, is highly effective in inhibiting bacterial infections. Moreover, it has been found that bacterial biofilms contain considerable amounts of GSH. Reducing the level of GSH can prevent its consumption of ROS and continuously deplete the GSH leaked from bacteria, significantly enhancing the effectiveness of PDT. Therefore, developing an integrated therapeutic system that combines PDT with GSH depletion could offer a new and promising paradigm for anti-biofilm infection. For example, Shi et al. coordinated Zr^4+^ ions with carboxyl groups of TCPP molecules to prepare Zr-TCPP MOF materials [39]. Leveraging the large specific surface area of MOFs and the presence of free carboxyl groups, they facilitated the loading of other metal ions such as Cu^2+^. This resulted in the preparation of MOF@Cu^2+^ composites (Figure 8A). Subsequent antibacterial experiments validated the effectiveness of this composite. The results showed that the loaded Cu^2+^ ions could effectively interact with GSH within the bacterial biofilm. Under laser irradiation at 638 nm, the porphyrin photosensitizer-generated ROS to disrupt the bacterial biofilm, while the reduction of GSH by Cu^2+^ minimized the biofilm’s resistance to oxidative stress (Figure 8B). Furthermore, researchers demonstrated that Cu^2+^ not only exhibits good biocompatibility but also promotes angiogenesis, thereby accelerating wound healing. The authors verified that the nanoplatform prepared in this system exhibited excellent antibacterial biofilm activity against Staphylococcus aureus both in vitro and in vivo (Figure 8B). This system not only provides a new approach for antimicrobial treatment and biofilm inhibition but also expands the phototherapeutic applications of porphyrin-based MOFs. It further substantiates that GSH depletion mechanisms can enhance PDT processes mediated by porphyrin-based MOFs.

## 10. Conclusions

In recent decades, porphyrin-based nMOFs have garnered extensive attention due to their excellent biocompatibility and superior photochemical properties [108,109,110]. The porphyrin-based nMOF therapeutic platform has become an effective drug delivery system for porphyrins and their derivatives, owing to its excellent stability and high photosensitizer loading capacity. A typical porphyrin-based nMOF comprises two main components: the porphyrin photosensitizer molecules and metal nodes. Much of the earlier research focused on the generation of ^1^O_2_ by porphyrin photosensitizers upon excitation by LED or laser light. As studies into the functionalization of metal ions and the tumor microenvironment have advanced, researchers have discovered that developing tumor microenvironment-responsive porphyrin-based MOFs through metal redox reactions can introduce new therapeutic mechanisms and significantly enhance PDT efficacy. For instance, multivalent metal ions such as Fe^3+^, Cu^2+^, and Mn^3+^ can not only coordinate with TCPP to form MOF materials for efficient intracellular delivery of hydrophobic molecules but also leverage their variable valence states to react with the excess GSH in cancer cells [34,35,36,37,38]. This reaction reduces the metal ions while preventing GSH from scavenging ROS generated during the PDT process. This review summarizes how Cu^2+^-, Fe^3+^-, and Mn^3+^-coordinated porphyrin-based nMOFs exhibit enhanced PDT processes through GSH depletion within cancer cells. These studies have ingeniously utilized all functionalities of the MOFs’ metallic and TCPP components. They achieve modulation of MOF photoreactivity to minimize damage to normal cells and exploit GSH-induced MOF structure disruption for TCPP release, thereby enabling fluorescence imaging and enhanced photon utilization efficiency. Moreover, the reduced metal ions resulting from GSH-induced valence changes can provide MR imaging capabilities, ensuring reliable targeted drug delivery. This summary holds significant importance as it provides a theoretical foundation for the development and biomedical applications of multifunctional, tumor microenvironment-responsive porphyrin-based nMOFs.

Despite the significance of these research efforts, detailed studies remain relatively sparse, indicating ample room for further exploration in this field. There are still several issues that require additional investigation and discussion. For example, comparative studies on the reaction rates of different metals with GSH; whether targeting specific organelles, which may have varying GSH concentrations, could optimize GSH depletion; and the limitations of using 640–660 nm wavelength light sources for treating deep-seated cancer cells. Additionally, some of the porphyrin-based MOFs discussed in this review require the framework to be disrupted to release metal ions for interaction with intracellular GSH, thereby facilitating its scavenging action. Consequently, highly stable porphyrin-based nMOFs may fail to participate in these reactions promptly due to their slower release rates. The large-scale production and clinical application of porphyrin-based nMOFs still face numerous challenges. In-depth research on their in vivo metabolic mechanisms is necessary to comprehensively evaluate their pharmacological and toxicological effects. Immunotherapy has been recognized as an effective approach for cancer treatment in recent years [111,112,113]. Given that GSH depletion itself may lead to changes in intracellular immune factors [114], combining GSH depletion with immunotherapy and PDT could potentially yield unexpected therapeutic outcomes. Addressing these questions is crucial for enhancing the preclinical application potential of porphyrin-based MOFs.

## Figures and Tables

**Table 1:** Synthesis of nMOFs and their GSH depletion mechanisms for enhanced antitumor applications.

Material	Structure		GSH Depletion Mechanism/Therapeutic Advantages	Refs
	Porphyrin	Nanoscale MOF		
Cu^2+^ for GSH depletion Au/Cu-TCPP(Fe)	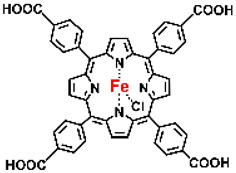	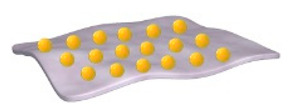	Au → glucose depletion → reduced GSH biosynthesis, Cu^2+^ + GSG → Cu^+^ + GSSG, Ferroptosis therapy,	[45]
Cu^2+^ for GSH depletion 2D Cu-TCPP	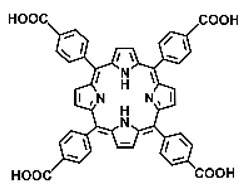	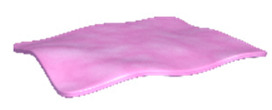	Cu^2+^ + GSG → Cu^+^ + GSSG, Russell mechanism, ^1^O_2_-induced tumor inhibition	[46]
Disulfide/thiol-induced GSH depletion Ce6@RMOF	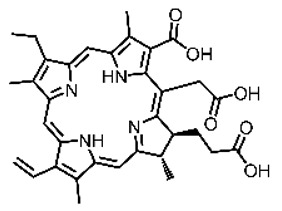	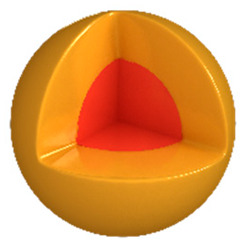	GSH + RS-SR → GSSG + RSH, PDT + Ferroptosis	[47]
Azo-induced GSH depletion MOF@Ce6	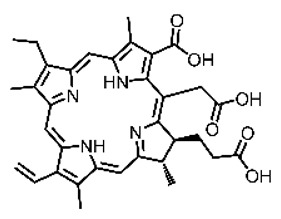	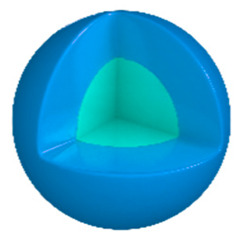	Azo → GSH and thioredoxin depletion, PDT, 4T1 cancer cells	[48]
Cu^2+^ for GSH depletion CCP	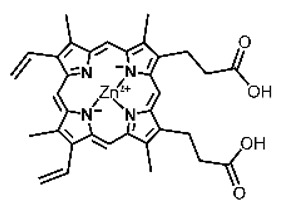	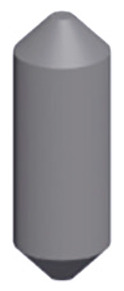	Cu^2+^ + GSG → Cu^+^ + GSSG, Heme oxygenase-1 activity suppression, CDT	[49]
Cu^2+^ for GSH depletion PCN-224(Cu)	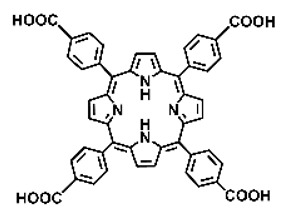	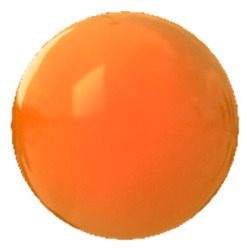	Cu^2+^ + GSG → Cu^+^ + GSSG, O_2_ generation, FL + MRI, chemodynamic and starvation therapy	[50]
Cu^2+^ for GSH depletion O_2_-Cu/ZIF8	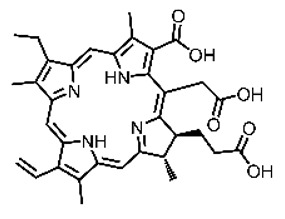	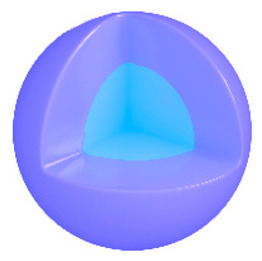	Cu^2+^ + GSG → Cu^+^ + GSSG, Cu^+^ + H_2_O_2_ → Cu^2+^ + ·OH, O_2_ replenishment, PDT + CDT	[51]
GSH adsorption to Zr^4+^ Zr-TCPP(Mn)	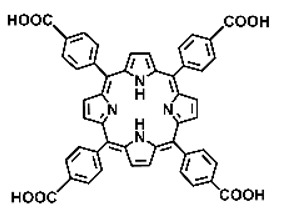	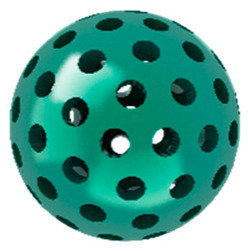	GSH depletion (adsorption to Zr^4+^), self-supply O_2_ Enhanced SDT + Ferroptosis	[52]

Abbreviations: FL (fluorescence imaging); MRI (magnetic resonance imaging).

**Table 2:** Porphyrin-based nMOFs and their GSH depletion mechanisms for enhanced PDT applications.

Material	Structure		GSH Depletion Mechanisms/Therapeutic Advantages	Refs
	Porphyrin	Nanoscale MOFs		
Mn(III)-TCPP MOFs	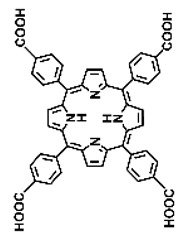	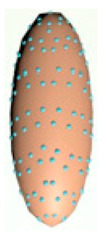	Mn^3+^ + GSH → GSSG + Mn^2+^, FL + MRI, 660 nm, 4T1 cells	[34]
Zr-TCPP MnFe2O4@MOF@PEG	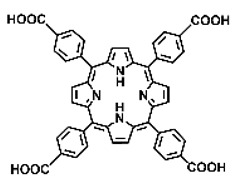	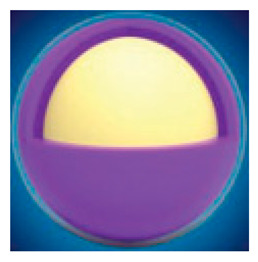	Mn^4+^ + GSH → GSSG + Mn^2+^, Fe^3+^ + GSH → GSSG + Fe^2+^, O_2_ generation, 660 nm, 4T1 cells	[35]
Fe-TCPP nMOFs@HANPs	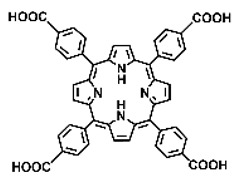	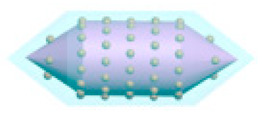	Fe^3+^ + GSH → GSSG + Fe^2+^, PDT + CDT, 635 nm, HA targeting, MR imaging, FL imaging, 4T1 cells	[36]
Zr-TCPP Cu^2+^-MOF HN@Cu-MOF	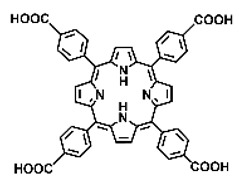	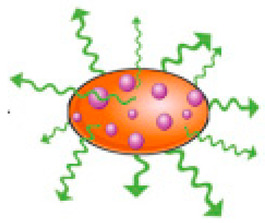	Cu^2+^ + GSH → GSSG + Cu^+^, Cu^+^ + H_2_O_2_ →·OH + Cu^2+^, Gas therapy (NO)+PDT, 4T1 cells	[37]
UCNPs PCN-224(Fe) UPFB	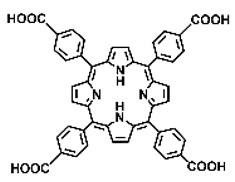	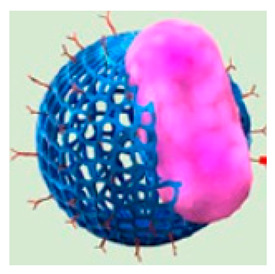	Fe^3+^ + GSH → GSSG + Fe^2+^, Fe^2+^ + H_2_O_2_ → OH + Fe^3+^, PDT + SDT + CDT, O_2_ generation, US/808 nm activation, U14 cells	[38]
Zr-TCPP MOF@Cu^2+^	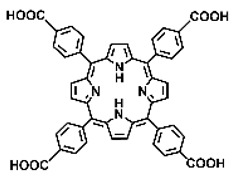	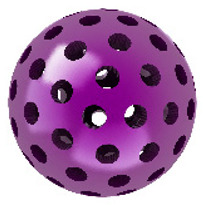	Cu^2+^ + GSH → GSSG + Cu^+^, 638 nm, PDT, biofilm eradication/wound healing	[39]
